# [Retracted] Homeobox B9 facilitates hypertrophic scar formation via activating the mitogen-activated protein kinase signaling pathway

**DOI:** 10.3892/mmr.2026.13906

**Published:** 2026-05-18

**Authors:** Qun Xie, Dandan Liu, Mosheng Yu, Xiaowei Wu, Yueqiang Zhao, Qiang Hu, Qi Wang

Mol Med Rep 16: 1669–1676, 2017; DOI: 10.3892/mmr.2017.6836

Following the publication of the above paper, it was drawn to the Editor's attention by a concerned reader that the western blot data shown in Fig. 5A and D had already appeared previously in an article written by different authors at different research institutes, which was published in the journal *Molecular and Cellular Biology*. Upon performing an independent analysis of the data in this paper, it also came to light that, concerning the histological images shown in Fig. 1, the ‘FN/HS’ data panel had also been previously published in an article written by different authors in the journal *Archives of Plastic Surgery*.

Owing to the fact that the abovementioned data in this article had already been published prior to its submission to *Molecular Medicine Reports*, the Editor has decided that this paper should be retracted from the Journal. The authors were asked for an explanation to account for these concerns, but the Editorial Office did not receive a satisfactory reply. The Editor apologizes to the readership for any inconvenience caused.

